# Supplementary material for: Efficacy and Safety of Tinzaparin in Prophylactic, Intermediate and Therapeutic Doses in Non-Critically Ill Patients Hospitalized with COVID-19: The PROTHROMCOVID Randomized Controlled Trial
Source: J Clin Med. 2022 Sep 24;11(19):5632. doi: 10.3390/jcm11195632 (PMC9571371; doi:10.3390/jcm11195632)
Supplement: Supplementary file 1 [file jcm-11-05632-s001.zip › jcm-1889079-supplementary.pdf]

CONFIDENTIAL

CLINICAL TRIAL PROTOCOL SUMMARY

Clinical trial evaluating optimal thromboprophylaxis and anticoagulant strategies in patients with COVID-19 pneumonia.

Multicenter, prospective, randomized, open study with parallel groups.

Brief Title: Trial Evaluating Optimal Thromboprophylactic and Anticoagulation strategies in patients with COVID-19 pneumonia.

Version: 2

Date: 20.01.2021

Code: PROTHROMCOVID.

Eudract number: 2020-004279-42

Principal Investigator - Coordinator: Dra. Nuria Muñoz Rivas, MD, PhD

PROTOCOL SUMMARY

**PROMOTER**	Fundación para la Investigación e Innovación Biomédica (FIIB) del Hospital Universitario Infanta Leonor y Hospital Universitario del Sureste
**TITLE**	Clinical trial evaluating optimal thromboprophylaxis and anticoagulant strategies in patients with COVID-19 pneumonia.
**EUDRACT NUMBER**	2020-004279-42
**CODE**	PROTHROMCOVID
**PRINCIPAL INVESTIGATOR-COORDINATOR**	Dra. Nuria Muñoz Rivas
**SCIENTIFIC COMMITTEE AND PRINCIPAL INVESTIGATORS**	**Scientific Committee**:<br>Dr. Nuria Muñoz Rivas<br>Dr. Juan Torres Macho<br>Dr. Jose Ángel Hernández Rivas<br>**Principal Investigators:**<br>Detailed in Annex IV
**CENTERS**	Detailed in Annex IV
**CRO**	S&H Medical Science Service<br>C/ Manuel Tovar, 43 Bajo Dcha – 28034 Madrid (España)
**ELECTRONIC CASE REPORT FORM DEVELOPMENT**	S&H Medical Science Service<br>C/ Manuel Tovar, 43 Bajo Dcha – 28034 Madrid (España)
**PHASE OF CLINICAL TRIAL**	III
**OBJECTIVES**	Evaluate the efficacy and safety of three doses of tinzaprin (prophylactic, intermediate and therapeutic) in hospitalized patients with COVID-19.
**STUDY DESIGN**	National study, multicenter, prospective, randomized, controlled, open, parallel group clinical trial. Patients will be randomized to:<br>Group A: tinzaparin 4500 UI/day;<br>Group B: tinzaparin 100 UI/kg/day;<br>Group C: tinzaparin 175 UI/kg/day
**STUDY POPULATION**<br>(Inclusion and Exclusion Criteria)	**<u>Inclusion Criteria:</u>**<br>1. Patients admitted to hospital with COVID-19 PCR and/or Antigens Test + SARS-CoV-2 infection or (presence of infiltrate compatible with Chest X-ray or TC).<br>2. Patients with, at least, one of the following evolution disease risk criteria:<br>-Sat 02<94%<br>-Need for oxygen therapy or pAO2/FiO2<300mmHg or estimated PaO2/FiO2 based on SpO2/FiO2<300 mmHg.<br>-DD>1000µg/L<br>-PCR >150mg/L<br>-IL6 >40pg/ml

	3. Age > 18 years<br>4. Weight entre 50 y 100 kg<br>5. After receiving oral and written information about the study, patient must give Informed Consent duly signed and dated before performing any activity related to the study.<br><br>**Exclusion Criteria:**<br>1. Patients who need mechanical ventilation (invasive or non-invasive), high flow nasal cannula or admission to ICU at the moment of randomization.<br>2. Current diagnosis of acute bronchial asthma attack.<br>3. History or clinical suspicion of pulmonary fibrosis.<br>4. Current diagnosis or suspicion of pulmonary thromboembolism or deep vein thrombosis.<br>5. Patients who need anticoagulant treatment due to previous venous or arterial thrombotic disease, or due to atrial fibrillation.<br>6. Patients with pneumonectomy or lobectomy.<br>7. Renal failure with Glomerular filtration <30 ml/min/1.73m2.<br>8. Patients with contraindication for anticoagulant treatment.<br>9. Congenital bleeding disorders.<br>10. Hypersensitivity to tinzaparin or UFH or some of its excipients.<br>11. History of heparin-induced thrombocytopenia.<br>12. Active bleeding or situation that predispose to bleeding.<br>13. Moderate or severe anaemia (Hb<10 g/dl).<br>14. Low platelet count < 80000/µl.<br>15. Patients with life expectancy less than 3 months due to primary disease evaluated by the physician.<br>16. Patients currently intubated or intubated between the screening and the randomization.<br>17. Pregnancy.
**MAIN VARIABLE**	Efficacy: Combined variable that includes reduction of suspicion of systemic thrombotic symptomatic events and/or need for mechanical ventilation and/or death at day 30 after randomization.<br>Safety: To determine the safety of different strategies of prophylaxis and anticoagulation in patients admitted to hospital with SARS-COv2 pneumonia.
**SECONDARY OBJECTIVES**	1. Reduction of suspicion of systemic thrombotic events (myocardial infarction, ischemic stroke, deep vein thrombosis, pulmonary thromboembolism confirmed by imaging tests).<br>2. Progression in the OMS Scale (worst situation during admission to hospital and hospital discharge).<br>3. Progression in the Acute Respiratory Distress Syndrome by criteria of PaO2/FiO2 or SpO2/FiO2.<br>4. Global survival day 14, 30 and 90.<br>5. Duration of hospital stay.<br>6. Orotracheal intubation<br>7. Duration of ICU stay.<br>8. Incidence of major bleeding.<br>9. Incidence of non-major bleeding clinically relevant.<br>10. Incidence of bleeding clinically relevant.

	11. Incidence of adverse reactions.<br>12. Changes in biochemical and haematological values from day 1 to 14 between groups.
**SAMPLE SIZE**	600 patients
**TREATMENT DURATION**	From randomization to hospital discharge.
**FOLLOW-UP**	90 days.
**RECRUITMENT ESTIMATED PERIOD**	Estimated date for inclusion of first patient: January 2021<br>Estimated date for inclusion of last patient: April 2021<br>Estimated date for complete follow-up of last patient: July 2021<br>The first results will be analyzed 30 days after the follow-up of the last patient.
